# Cross Layer Adaptation of Check Intervals in Low Power Listening MAC Protocols for Lifetime Improvement in Wireless Sensor Networks

**DOI:** 10.3390/s120810511

**Published:** 2012-08-02

**Authors:** Soledad Escolar, Stefano Chessa, Jesús Carretero, Maria-Cristina Marinescu

**Affiliations:** 1 Computer Science Department, University Carlos III of Madrid, Avda. Universidad 30, Madrid 28911, Spain; E-Mails: jesus.carretero@uc3m.es (J.C.); mariacristina.marinescu@gmail.com (M.-C.M.); 2 Computer Science Department, University of Pisa, Largo B. Pontecorvo 3, Pisa 56127, Italy; E-Mail: ste@di.unipi.it; 3 ISTI-CNR, via G.Moruzzi 1, 56124 Pisa, Italy

**Keywords:** communication latency, synchronization, preamble sampling protocols, adaptive scheduling, wireless sensor networks

## Abstract

Preamble sampling-based MAC protocols designed for Wireless Sensor Networks (WSN) are aimed at prolonging the lifetime of the nodes by scheduling their times of activity. This scheduling exploits node synchronization to find the right trade-off between energy consumption and delay. In this paper we consider the problem of node synchronization in preamble sampling protocols. We propose Cross Layer Adaptation of Check intervals (CLAC), a novel protocol intended to reduce the energy consumption of the nodes without significantly increasing the delay. Our protocol modifies the scheduling of the nodes based on estimating the delay experienced by a packet that travels along a multi-hop path. CLAC uses routing and MAC layer information to compute a delay that matches the packet arrival time. We have implemented CLAC on top of well-known routing and MAC protocols for WSN, and we have evaluated our implementation using the Avrora simulator. The simulation results confirm that CLAC improves the network lifetime at no additional packet loss and without affecting the end-to-end delay.

## 1. Introduction

A Wireless Sensor Network (WSN) [1] is an ad-hoc network specialized in environmental monitoring that is composed of autonomous, cooperating, small-sized nodes connected through wireless links, and a special node, the sink, that can forward data from the nodes to external users. Each node in a sensor network is equipped with a processor, one or more sensing units, and a radio transceiver. It is powered by an embedded battery [2], which is, in general, the sole source of energy. This means that the lifetime of the nodes is limited, and there is great emphasis on the efficient use of energy to prolong battery life.

Among the low energy strategies in WSN, the approaches operating at the MAC layer are without doubt the most numerous. It is well known [3] that the radio transceiver is the most energy-consuming component of a node. The MAC layer is responsible for managing this component by scheduling the times when the node must turn its radio on and off. This makes the MAC layer the main responsible component for saving energy. This idea is the basis of preamble sampling MAC protocols, which periodically toggle the radio from sleep to active state to check the channel for incoming packets. If there is no activity on the channel the radio is shut off to keep the activity period short and thus save energy. For this approach to work well, the time when the radios are activated must match for each pair of communicating nodes. Otherwise, if a node transmits a packet later than the originally scheduled time, the receiving node may not be listening, which would cause the loss of the packet followed by retransmission. Conversely, a node may listen to the channel when no one is sending (idle listening). To solve this problem, preamble sampling protocols use some form of synchronization among 1-hop neighboring nodes.

Most of these strategies are based on sending long preambles or check intervals. The first approach ensures that the preamble can be detected in the receiver node in order to maintain the radio on as long as necessary to receive the data. The second approach consists of computing a check interval that is shared for neighboring nodes and which enables the nodes to synchronize the times at which they switch their radios on and off. Both these approaches may negatively affect communication latencies. Specifically, nodes may experience delays in the transmission and reception of the packets as a consequence of the medium contention and of the strict activity periods. Moreover, in these protocols the radio scheduling is generally static, which means that it is not updated in the presence of communication delays [4–8]. This results in packet loss and packet retransmission, which increases the energy consumption. This problem becomes worse in multi-hop scenarios, where the communication delays increase as they are propagated along the path from the sender to the sink.

In this paper we propose Cross Layer Adaptation of Check intervals (CLAC), an approach that is concerned with the trade-off between communication delays and energy consumption which occurs in preamble sampling protocols based on check intervals. The main goal of CLAC is to reduce the energy consumption of the nodes without significantly increasing delay, by adjusting appropriately the nodes scheduling. CLAC exploits information extracted from the application, routing, and MAC layers to estimate the communication delays that affect a packet traveling along a multihop path. It then uses this estimation to recompute and update the value of the check intervals at each hop such that each node can adapt to the expected packet arrival time according to the accumulated delays along the paths. We have implemented and evaluated CLAC using the TinyOS/MicaZ platform on top of the BoX-MAC data link protocol and Collection Tree Protocol (CTP) routing protocol. As compared to CTP/BoX-MAC, CLAC improves the network lifetime of the communicating nodes without increasing the end-to-end delay and without additional packet loss. The extension of CLAC to other low power listening protocols is straightforward, and it is object of ongoing work.

The remainder of the paper is organized as follows. Section 2 presents the related work dealing with communication latencies suffered by nodes as a consequence of operating in (low) duty cycles to save energy and describes the protocols that we used in our evaluation. In Section 3 we formulate the problem scenario. Section 4 describes CLAC and discusses how it computes the network latencies which occur in preamble sampling-based MAC protocols to appropriately synchronize the nodes. Section 5 presents our evaluation and results, which are compared to the ones obtained by a preamble sampling-based MAC implementation (BoX-MAC). Finally, Section 6 draws the conclusions and suggests future work.

## 2. Related Work

Surveys in the literature [9–12] have identified four main sources of energy waste at the MAC level: (1) idle listening, which occurs when a node listens the channel but no one is sending; (2) collisions, which occur when two or more nodes transmit at the same time, causing packet loss and possibly subsequent retransmissions; (3) overhearing, which occurs when a node listens for a packet targeted to another node; and (4) overhead due to the control messages that support MAC/routing operations. These surveys also provide an insight into MAC protocols for WSN.

In a recent work [13] the authors provide a classification of MAC protocols in three categories:

- *Scheduled protocols* are synchronous protocols based on Time Division Multiple Access (TDMA). Here, each node must know the time slots assigned to its neighbors in order to wake up (*i.e.*, turn its radio on) during these transmission slots. These approaches require an ad-hoc scheduling of the nodes' activity periods based on their communication needs. Furthermore, communication delays due to this schedule can be limited only when the network traffic is low.
- *Asynchronous protocols* do not manage a common schedule for all the nodes, rather, each node decides independently its radio activation period. To ensure that the communication among two nodes takes place, even if the nodes have chosen different radio activation periods, these protocols use strategies such as *preamble sampling* or *receiver-initiated communications*. Asynchronous protocols are particularly interesting in WSN since they do not have overhead due to node synchronization and they may enable very low duty cycles. For these reasons their energy consumption can be lower than in other approaches. On the other hand, they can incur communication delays caused by the lack of synchronization of the nodes and the channel access contention.
- *Protocols with Common Active Periods* can be considered hybrids between the two former approaches. In these protocols, the nodes share the scheduling time of the radio activity periods (which are typically longer than those used by other approaches). A node can send data at any time during its radio activity period. Channel contention is typically managed by a handshake protocol (for example based on RTS/CTS [14]). In these strategies, the overhead due to the maintenance of the common scheduling and the longer duration of the active periods tend to increase the energy consumption. However, since these protocols need a certain degree of synchronization among nodes, they can reduce the contention delays as compared to asynchronous-like approaches.

In this work we focus on asynchronous protocols based on preamble sampling. A survey of these approaches is provided in [13]. Next, we provide a more detailed explanation of the operation of these protocols.

### 2.1. Preamble Sampling-Based MAC Protocols

The idea of MAC protocols based on preamble sampling is that every node toggles periodically the radio from sleep to active mode, with the aim of sampling the channel to detect incoming packets. The interval between two consecutive samplings of the channel is the sampling interval, also called *check interval*, and its length is *τ_check_*. If during the sampling there are no packets to be received, the node toggles the radio to sleep. Otherwise, the node keeps the radio on for the time necessary to receive the incoming packets, and then it turns the radio off. The goal of this strategy is to reduce the energy consumed by idle listening. This concept, with small differences, was proposed independently by two authors (Hill *et al.* [15] and El-Hoiydi [5]), and given two different names: *Low Power Listening* (LPL) and *preamble sampling*. Both names are currently used in the literature to refer to the same concept. Hereafter we use them interchangeably.

**Figure 1.**
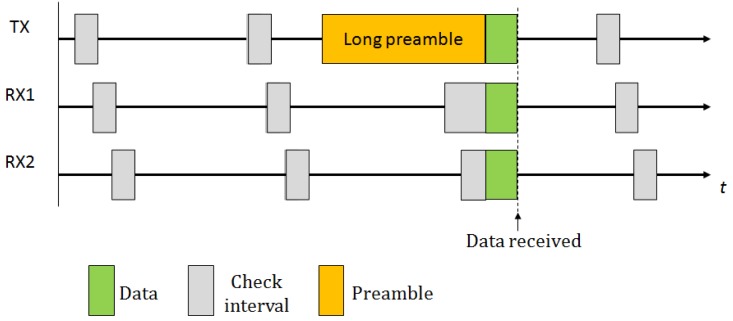
Basic preamble sampling scheme.

To ensure that a packet is correctly received by its recipient, the LPL sender transmits a very long preamble immediately before the actual packet. This preamble spans the whole check interval to ensure that, when the receiver starts its check interval, it detects the preamble and remains in active mode for the time necessary to receive the packet. Figure 1 illustrates the strategy used in this technique. The protocol presents some disadvantages, such as an excessive amount of energy consumption as compared to approaches based on check intervals as well as a high overhead as a consequence of the transmission and reception of very long preambles. The overhearing is also very high, since the packet has to be completely received to determine its destination. Another limitation of these approaches is that they cannot be applied to all types of networks, specifically, those networks where the preamble is fixed and limited to a few bytes, such as IEEE 802.15.4-compliant networks [16,17] (note that, however, to address the latest point, there exist other approaches based on sending short preambles such as X-MAC [18]). For these reasons, many variants and modifications of the original protocol have been proposed. In particular, we highlight WiseMAC [6], an energy efficient MAC protocol based on synchronized preamble sampling. WiseMAC considers infrastructure networks and assumes multiple access points that are usually energy unconstrained. WiseMAC focuses on the downlink problem: the communication from the access point to the nodes. The basic idea behind WiseMAC is to reduce the length of the preamble based on the knowledge of the receiver's sampling schedule, which is piggybacked into the acknowledgment packets. Thus, the sender starts to transmit the preamble just a short time before the wake-up time of the receiver. Another approach is VLPM [19] that considers both the downlink and uplink problem for Wireless Body Area Networks (WBAN). In VLPM, the nodes are equipped with both a low-power transmitter (employed for sending wake-up packets before sending data) and a receiver (employed for continuous monitoring of the activity on channels).

### 2.2. Delay-Aware MAC Protocols

Although LPL protocols reduce the energy consumption by reducing the idle listening periods, they may introduce longer end-to-end communication latencies as a consequence of the medium contention and of the strict duty cycles governing the activity of the nodes. Low duty cycles increase the latencies, since the nodes can transmit and receive packets only during the window of time scheduled for their duty cycle. Thus, senders may avoid the transmission if the channel is busy, and receivers may become too burdened to be able to manage the incoming traffic with their current LPL configuration.

Many works in literature study whether energy-efficient duty cycles may be maintained without affecting quality of service (QoS) parameters (throughput, latency, reliability). One approach is to dynamically adjust the duty cycle based on the traffic load and/or topology information. Thus, some authors propose to increase or decrease the sleep interval under certain network conditions. For example, in BoostMAC [20] the nodes sample the medium with a periodicity of *t*. If the sampling node finds the medium idle, it increments *t* by *t_add_*, up to a maximum *t_max_*; otherwise, it divides the current sampling period *t* by *t_dec_*. MaxMAC [21] uses the number of packets received as a metric to react to the network congestion. This protocol adapts to the current traffic by switching between four states (“basic”, “extra”, “more-extra”, and “CSMA”) that represent congestion levels. The transitions between states occur based on comparing the packet rate with thresholds defined for each state: if the threshold is passed, the protocol halves the sampling interval and switches to a state representing higher congestion (up to “CSMA”); otherwise the protocol doubles the sampling interval and switches to a state representing lower congestion (down to “basic”).

SCP-MAC [22] uses adaptive channel polling to detect packets traveling over multiple hops. There are also other protocols that balance the load based on routing information. One example is Energy Aware Adaptive LPL (EA-ALPL) [23], which is built on top of B-MAC. It dynamically selects its own listening and transmission modes among those provided by B-MAC. The selection is done according to the duty cycle and number of descendants in the routing tree. Another approach is Adaptive Staggered sLEEp Protocol (ASLEEP) [24], which uses the number of received packets and the inter-arrival time to compute the length of the activity period in the next communication interval. In order to synchronize neighboring nodes, each node sends its own scheduling to its neighbors by means of beacon messages.

All the approaches discussed above search for a balance between energy and throughput requirements, and, as a side effect, may reduce communication delays. Other studies analyze the delays for different MAC protocols. For example, [25] adopts a TDMA protocol that assigns receiving slots to sleeping nodes. Under this scenario the authors investigate the delay-efficient sleep scheduling for two different communication patterns, *i.e.*, when every pair of nodes is equally likely to communicate and when it is not so. Using graph theory the authors formulate an equation for computing the delays and demonstrate that the minimization of delays for each pair of nodes in the graph is NP-hard. Then, they analytically find optimal solutions for two specific topologies: trees and rings. Finally, they suggest several heuristics for other arbitrary topologies. DW-MAC [26] describes the design and implementation of a new CSMA-based protocol, which is designed for reducing the packet delivery latencies in applications that have high traffic load. In [27] the authors consider an anycast communication scenario and they propose a probabilistic model to find an optimal solution for the delay minimization problem that maximizes the network lifetime. In [28] the authors investigate nodes synchronization in interruptible LPL protocols, *i.e.*, LPL protocols that use an acknowledgment packet to interrupt the sender in its continuous transmission of data. In this paper, the authors compute for each node its new check interval by adding a delay *T_S_* that is computed as the difference between the wake-up times of the communicating nodes. Further research of the same authors [29] propose to adjust the transmitter and receiver schedules from a pool of interruptible LPL protocols to meet the dynamics of the node and the network conditions. In another work [30] these authors study the dynamic adaptation of the duty cycle in interruptible LPL protocols. Firstly, they propose to adapt the duty cycle by increasing the check interval by 100 ms when five consecutive packets are successfully sent to the destination and by decreasing the check interval by 250 ms when the packet does not achieve its destination. Secondly, they optimize the check interval by using control theory in order to find a good balance between energy savings and packet delivery successes.

In [31] we present preliminary work adjusting the check intervals of LPL protocols based on the communication delays suffered by the packets traveling along multi-hop paths. The present work describes improvements to the adjustment mechanism for tree topologies by taking into account the case of multiple sources; it also presents comparison results between CLAC on top of CTP/BoX-MAC and CTP/BoX-MAC alone, from the point of view of the network lifetime, connectivity, end-to-end delay and packet loss.

In contrast to [28,29] which considers single-hop scenarios, CLAC computes the delays in multi-hop scenarios. A multi-hop scenario is also considered in [30], in which the delay is estimated based on local observations (e.g., number of packets successfully sent and received) while in CLAC it is assumed that the packet transmission rate is known (as parameter of the application running on the sensors), and the delay is estimated based on the positions of the nodes within a path. Thus, CLAC may easily adapt to changes in the network topology while in other works it is assumed that the topology rarely changes.

### 2.3. Background

In this subsection we describe BoX-MAC and CTP, the MAC and routing protocol implementations that we use in our work.

#### 2.3.1. BoX-MAC

**Figure 2.**
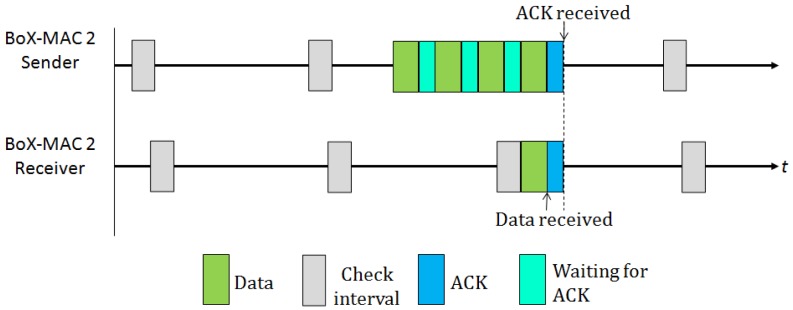
BoX-MAC-2 Tx/Rx scheme.

Some existing MAC protocols exploit implicit synchronization information to eliminate long preambles. Specifically, BoX-MAC [8] is an example of interruptible LPL protocol that disseminates the check intervals of the nodes by piggybacking, so that the time of activity is shared among neighboring nodes. BoX-MAC builds upon two successful MAC protocols: B-MAC and X-MAC. B-MAC [7] is a classical preamble sampling protocol; X-MAC [18] is a strobed preamble approach that allows the receiver to interrupt the long preamble as soon as it wakes up and determines that it is itself the intended recipient. For this reason, the address destination is also included in the preamble. In BoX-MAC a sender does not transmit a long preamble, rather, it repeatedly transmits the same data packet. The reception of the packets is done similar to the reception in B-MAC: once the receiver turns its radio on, it first examines whether there is an ongoing transmission, then it waits for the packet header in order to check whether it is itself the recipient of the packet. If that is the case, it keeps the radio on to receive the packet and immediately after receiving, it sends an acknowledgment (ACK) to the transmitter. Once the transmitter receives the ACK it stops retransmitting, as shown in Figure 2. Currently there exist two versions of BoX-MAC: BoX-MAC-1 that uses a predominantly physical-layer sampling approach, and BoX-MAC-2 that uses a predominantly link-layer packetized approach. The fact that BoX-MAC can be implemented on standard radio hardware makes it very appealing and, for this reason, this is the default implementation in TinyOS.

Although BoX-MAC reduces the energy consumption by 30% to 50% compared to other existing approaches, the latencies and the channel contention along a path increase linearly with the number of nodes on the path. In this work we investigate the impact of the network delays on the energy consumption and throughput in BoX-MAC-2.

#### 2.3.2. Collection Tree Protocol

Collection Tree Protocol (CTP) is a best-effort, multi-hop routing protocol designed for low power and low data rate WSN. For each node in the network, CTP proactively maintains a spanning tree rooted in the node itself that minimizes the cost of the path from any node to the root. The *cost* of a link is evaluated by using the ETX (Expected Transmissions) metric [32,33], which is the estimation of the number of transmissions required to successfully transmit a packet along the link.

Every node maintains a routing table which records the neighbors' identifiers and their cost to the root. To build this table, all the nodes in the network exchange beacon messages containing their cost to reach the root.

The purpose of CTP is to achieve robustness and reliability, and the results show that it successfully achieves high delivery packet ratios ranging between 90.5% and 99.9% [34]. However, to obtain this performance, CTP burdens the network with control packets (ACK, beacons, *etc.*). The cost of dealing with the dynamics of real networks (e.g., loop detection, packet loss, temporary lost of connectivity) generally results in higher energy consumption.

## 3. Problem Formulation

We consider a WSN executing a sensing task, where the nodes operate in periods or *rounds* whose duration is determined by their duty cycle. At each round each sensor samples the environment and produces a new data item that it sends to the sink, possibly along a multi-hop path. This behavior is typical of many WSN protocols, such as, for instance, Directed Diffusion [35].

As in most WSN applications, in our scenario the nodes are equipped with batteries that sustain completely their activities. Hence, an efficient use of the energy is of utmost importance in order to prolong the lifetime of the nodes.

One of the most energy consuming components of a node is the radio. A radio interface has at least four states: idle, ready, Tx (transmission mode), and Rx (reception mode). Every state has a different energy consumption, which depends on the particular model and manufacturer of the node: In idle mode the energy consumption is very low, and in the rest of the states the energy consumption can be considered similarly high. To save energy, nodes need to keep the radio in idle mode as much as possible. This is the approach usually used in MAC protocols for WSN. On the other hand, while a node is in idle mode, the radio cannot receive nor send packets and the node behaves as if it was disconnected from the network.

With the goal of saving energy in mind, the application manages a duty cycle that reflects the level of activity of the node. All operations that a node executes must fit in its duty cycle. In our approach we assume that the duty cycle of all of the nodes is identical; the MAC layer uses it to compute the check intervals which are then communicated to the neighboring nodes in order to share the same information.

Network lifetime is commonly defined as the time elapsed between the beginning of the network activities and the time at which the first node of the network depletes its battery. We adopt this precise definition in this work. Note that, however, there exist other definitions for the network lifetime that may also apply. Let *B* be the initial energy at each node battery; the lifetime *l*(*i*) of a node *i* measured as the number of rounds can be expressed as:

(1) $$l(i)=\frac{B}{E(i)}$$

where *E*(*i*) denotes the energy consumed by node *i* in a round. We are assuming that a node is alive as long as it performs all assigned functions. The lifetime of the network *L* is then:

(2) $$L={\text{min}}_{i}\{l(i)\}$$

Let us consider a WSN composed of *n* nodes, *i*_1_ to *i_n_*. Each node can communicate directly with its neighbors, *i.e.*, with the nodes within its radio range. For the sake of simplicity we assume that if *i_j_* is in range with *i_k_* then *i_k_* is also in range with *i_j_, i.e.*, communication links are bidirectional. A node may also communicate with a remote node, *i.e.*, a node which is not within its radio range, by a multi-hop path that consists of the set of nodes on the path. Let *path*(*i*_1_, *i_m_*) = 〈*i*_1_, *i*_2_, …, *i_m_*〉 be an *m*-hop path connecting nodes *i*_1_ and *i_m_*, and let *i_k_* be a node on this path. Then *order*(*i_k_*) denotes the place occupied by *i_k_* in the path, *i.e., order*(*i_k_*) is the hop distance between *i*_1_ and *i_k_*. This relation ensures that *order*(*i*_1_) ≤ *order*(*i_k_*) ≤ *order*(*i_m_*). Note that the function *order*(*i_k_*) within the *path*(*i*_1_, *i_m_*) returns a number between 0 and *m* − 1. This function is later used in Equation (4).

Routing decisions regarding the nodes on the path from a sender *i*_1_ to a receiver *i_m_* are left to the routing protocol, in our case CTP. The nodes use BoX-MAC as the underlying data link protocol. This implies that the nodes along the path wake up to sample the channel after each check interval expires. At this time, they may either receive the neighbor's packets and forward them to the parent, or initiate a transmission. It can still happen that some nodes activate their radios unnecessarily, since the communication rate required by the application can be much bigger than the check interval. (The check interval is typically in the order of milliseconds, while the communication rate can be in the order of seconds and even minutes.) Under perfect synchronization, the sender and the receiver wake up at the same time and thus avoid that BoX-MAC retransmits packets (because the ACK will be sent immediately). However, under realistic network conditions, packets travel along multi-hop paths towards the destination and accumulate delays at each hop. These delays are due to several reasons: (1) channel contention due to high traffic loads and small check intervals, which causes BoX-MAC to retransmit a large number of packets; (2) node processing due to packet forwarding and sensing activities; (3) protocol control packets that increase network load; (4) packet transmission times which may not match the check intervals of receivers.

**Figure 3.**
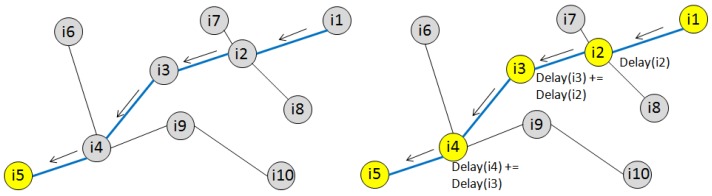
On the left, the routing protocol selects the path to connect two remote nodes *i*_1_ and *i*_5_ through the nodes *i*_2_, *i*_3_, and *i*_4_. On the right, nodes experience communication delays which are propagated down along the path from the source to the destination node.

Figure 3 shows, on the left hand side, a simple scenario where *i*_1_ sends data to a remote receiver *i*_5_.The arrows show the path between the two nodes as computed by CTP. The right hand side shows the delays incurred as data propagates from *i*_1_ to *i*_5_, and how these are accumulated during the multi-hop transmission.

**Figure 4.**
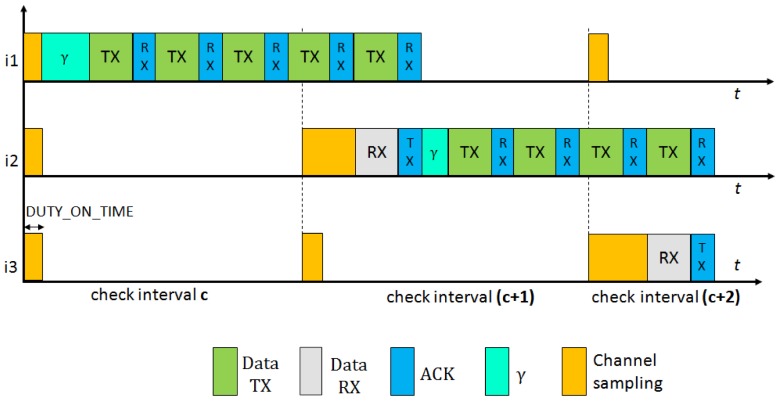
BoX-MAC behavior with communication delays.

In Figure 4 we present the behavior of BoX-MAC protocol, where nodes can incur communications delays. As explained in Section 2.3.1, a BoX-MAC node keeps transmitting a packet until it receives an acknowledgment packet from the receiver. Let *γ* be the delay introduced by node *i_k_* in the path (we define more precisely this delay in Section 4). In the figure, during the check interval *c*, node *i*_1_ introduces a delay before sending the packet and propagates it to the next hop in the path *i*_2_, which cannot receive the packet correctly. When *i*_2_ forwards the packet to the next hop *i*_3_ (note it is common that this happens in the next check interval), it introduces a new delay. Since *i*_3_ cannot receive the packet at the check interval *c* + 1 because the accumulated delay spans its time of channel sampling, *i*_2_ continuously retransmits the packet during the sleep time of *i*_3_. When *i*_3_ awakes in the next check interval *c* + 2, it receives the packet and sends an acknowledgment packet to *i*_2_, which stops the retransmission. Thus, the reception of the packets could be shifted to the next rounds of communication. To deal with these communication delays the check intervals need to be adjusted in each hop in order to match the time to turn the radio on in the sender and the receiver.

## 4. Cross Layer Adaptation of Check Intervals

We propose Cross Layer Adaptation of Check intervals (CLAC), an approach which consists in exploiting cross-layer information to adapt the check intervals according to the delays incurred by packets along a path. This technique allows us to maintain the duty cycle of the nodes without introducing additional energy costs. The basic idea is to improve synchronization among the nodes and, as a consequence, use energy more efficiently.

CLAC exploits information from the application, routing, and MAC layers. At the application layer the programmer statically configures a duty cycle (DC) representing the activity level of the nodes. Based on DC, the MAC layer configures the check interval, *τ_check_*; For instance BoX-MAC computes *τ_check_* as follows:

(3) $$\tau_{\text{check}}=\frac{\left(\text{DUTY\_ON\_TIME})\times (100-DC\right)}{\left(DC\right)}$$

where DUTY_ON_TIME is a constant that stands for the minimal time the radio is kept on in each activation, and DC is expressed as the percentage of activity with respect to the length of the total period. DC represents the minimum time of activity regardless of the network conditions. The maximum time of activity depends on the traffic load and therefore it is a priori unknown (BoX-MAC maintains the radio on when it detects channel activity). Equation (3) shows the inverse relationship between DC and *τ_check_*: a higher DC implies a smaller *τ_check_*. DC is established statically according to the frequency with which the application operates data sampling and transmission. For the sake of simplicity we assume that DC is the same for all of the nodes.

CLAC also exploits the information about the route from a node *i_j_* to a node *i_k_* as computed by CTP. Specifically, CLAC uses information about the position occupied by each node within the path to estimate the delay accumulated by a packet traveling along the path in each hop between *i_j_* and *i_k_*, then it recomputes *τ_check_* at each hop according to this delay. In our approach the delays are adaptive because routes can dynamically change, which implies a different node ordering. In multi-hop scenarios, as those addressed in this paper, each hop in the path introduces a certain delay, *γ*(*i_k_*), which stands for the total delay (including transmission time) experienced by a packet at the node *i_k_* within the path from *i_1_* to *i_m_*. The delay experienced by a packet along the path between *i_j_* and *i_k_* is the sum of the delays introduced by every one of the nodes that compose the path. The discussion of the value taken by *γ* is deferred to Subsections 4.1 and 4.2. Assuming that we know the value of *γ*, the total delay of a packet at a node *i_k_* is:

(4) $$D(i_{k})=\sum_{i_{j}=1}^{\text{order}(i_{k})}\gamma (i_{j})$$

where *D*(*i_k_*) represents the delay accumulated by a packet before reaching node *i_k_* within the path(*i*_1_,*i_m_*). Note that *D*(*i_k_*) increases with the number of hops between *i*_1_ and *i_k_*. Thus, the first node *i*_1_ in the path generates the packet and introduces a delay that is propagated to the second hop, *i*_2_, which is affected by this delay. *i*_2_ propagates both this delay as well as the delay introduced by itself further down to node *i*_3_. The delay for receiving the packet in *i*_3_ is the sum of all of the delays of the previous hops (in our case *i*_1_ and *i*_2_). In general, node *i_k_* must update its check interval as follows:

(5) $$\tau_{\text{new\_check}}=D(i_{k})+\tau_{\text{check}}$$

This update slightly shifts the current *τ_check_* of node *i_k_* by a value *D*(*i_k_*) to obtain its next check interval. It is important to note that increasing the check interval involves a slight decrease in DC, which in turn could lead to packet loss. Packet loss does not happen if the next packet reception fits in the interval given by [*τ_new_check_, τ_new_check_* + DUTY_ON_TIME]. This is the time during which the radio is kept in receive mode within each check interval. If the packet arrives at time within this interval, the BoX-MAC protocol extends the duration of the activity period to guarantee the complete reception of the packet regardless of the time of reception of a packet, called *τ*. Otherwise, if *D*(*i_k_*) > DUTY_ON_TIME, then the packet is lost.

**Figure 5.**
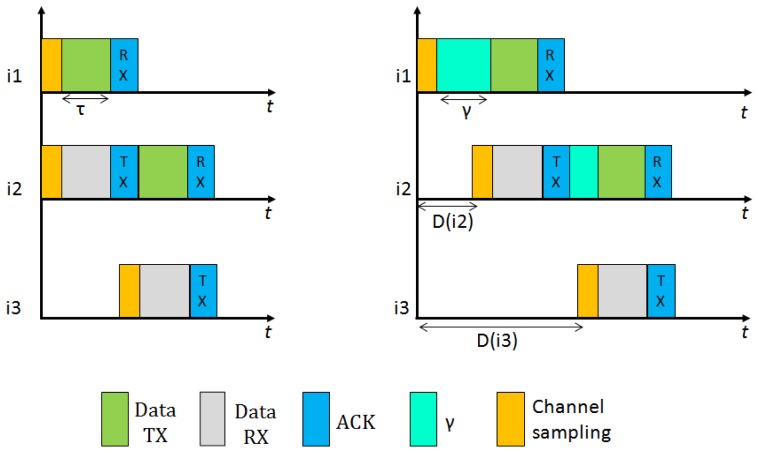
Behavior of CLAC in two different scenarios: on the left, the ideal case with no contention delays. On the right, a realistic case where delays are introduced at each hop. CLAC adjusts the next check interval in the receivers. In the figure *τ* stands for the time of transmission/reception of a packet of length *s* on the medium.

Consider the BoX-MAC behavior presented in Figure 4, where the reception of the packets is shifted to the next round of communication. CLAC can solve this problem by adapting the check intervals of the nodes as shown in Figure 5. We show two possible situations: On the left, an ideal situation where the nodes do not incur contention delays. CLAC adapts the check interval of each node to match the time to send and receive each packet. On the right, a more realistic scenario where network delays do exist. In this case, CLAC shifts the check interval of each node according to the delay introduced by the packet in order to wake up precisely at the packet arrival time. We can ensure that the packet is successfully received since the radio is maintained on during the time needed to complete its reception.

As indicated, CLAC uses *γ*(*i_k_*) to express the delay experienced by a packet at the node *i_k_*. This value is specific to each node and it depends on several factors: the network topology, the duty cycle, the frequency with which the application generates traffic, *etc.* We have analytically computed the value of *γ* by analyzing two different multi-hop scenarios: a linear and a tree-based data collection topologies.

### 4.1. Linear Topologies

In this topology the forwarding nodes are connected to exactly two neighbors: one child and one parent node. Each node receives packets from its child and forwards them to its parent to reach the destination node. Since the communication delays due to medium contention are related to the number of neighbors, for this particular topology we can estimate the value of *γ* to be approximately constant for each node on a path. We define *γ* in terms of the check interval and a parameter *p* that expresses the fraction of *τ_check_* by which the check interval should be shifted (to bring it forward or backward):

(6) $$\gamma =p\times \tau_{\text{check}}/100$$

For instance, given a check interval of 200 milliseconds, if *p* takes the value of 5, then the delay introduced by each packet at each node will be of 10 units of time (expressed in the same units that the check interval). The delay introduced by a packet at node *i_k_* depends on its position within the path and increases linearly with the number of hops. This approach makes sense for linear topologies where each node redirects the data to one single neighbor.

### 4.2. Tree-Based Topologies

Tree-based topologies are widely used in many WSN applications. In a tree topology each forwarding node has a number of children which is not necessarily the same for all of the nodes. The intermediate nodes forward all the incoming packets from their children to the single parent to reach the destination node. In many WSN applications which use a collection tree, nodes typically use filters to select the sensed data that needs to be transmitted to the sink. For example, in Directed Diffusion, all nodes sample the environment at the same given rate in order to detect a specific event. When a node detects the event, it starts transmitting any relevant data to the sink at a rate that corresponds to the sampling period. This means that in general not all the nodes produce data, but rather a subset of them, which we call sources. The sources emit data that travels along a path in the tree up to the sink (which in these cases is the root of the tree). From the point of view of the individual source, the data traveling along the path to the sink behaves as in the case of linear topologies. However, packets from different sources generally meet at some intermediate nodes in the tree and from that point on they follow the same path.

Consider the case in which a relay node *i_k_* receives data produced by different sources that can be either *i_k_*'s direct children or nodes in the subtree rooted at *i_k_*. All sources emit packets according to the same duty cycle, and therefore, the same period. If the packets emitted by the sources arrive at *i_k_* at different times, for instance if the sources are at different depths in the tree, the network behaves similar to a linear topology. If the packets from two or more sources arrive at *i_k_* at instances close in time they may experience additional delays due to channel contention. In these cases, the delay defined at *i_k_* should be configured to have *i_k_* ready for the fastest packet. The delay of the node will therefore be defined by the same formula as in the case of linear topologies: *γ*(*i_k_*) = *p* × *τ_check_*/100. It is important to remember that, after a packet is received by *i_k_* there may be others that are delayed due to channel contention. For this reason *i_k_* must keep its radio on for a period after receiving a packet, a behavior already present in BoX-MAC. Algorithm 1 shows the pseudo-code for CLAC.

Section 5 evaluates the delays for different values of *p* and for different network topologies.


**Algorithm 1** CLAC algorithm
 Configure *p   # MAC layer info* Configure *DC   # MAC layer info* Compute *t*_check=DUTY_ON_TIME × (100-*DC*)/ *DC* Obtain all *routes* crossing node *i    # Routing layer info* D(i)=∞ **for all** (*r* ∈ *routes*) **do**  Compute *order*(*i*) in *r*(*j, k*), *j*=source,*k*=sink  Compute delay *D_r_* (*i*)=*order*(*i*) × *p* × *t_check*/100  Compute *D*(*i*) = *min*(*D_r_*(*i*),*D*(*i*)) **loop**  Compute *t_new_check*= *t_check* + *D*(*i*)


## 5. Evaluation

We implemented CLAC on top of TinyOS [4] and we used available implementations of CTP and BoX-MAC-2 as the routing and MAC protocols. We also implemented an application where each node generates a 40-byte length data packet at constant intervals of 10 seconds and forwards incoming packets from children to the sink. This application is implemented on top of CLAC and uses the routing and MAC information to adapt the check intervals as discussed in this paper. We developed CLAC specifically for the MicaZ [36] platform and we tested it on linear and tree-based topologies, for which we evaluated networks of different sizes and densities.

To represent meaningful variations in the node activity we configured the application with different duty cycles. Table 1 shows in the first column the values of DC and in the second column their corresponding check intervals according to Equation (3). The subsequent columns show the delay that is accumulated by each hop as well as the value of the next check interval (*τ_new_check_*) with *order*(*i_k_*) = 1 given different values of *p* (−1, 1, 5, and 10). These values are the same as those that we used to compute the accumulated delay at each hop as explained in the previous section. Note that the values of *τ_new_check_* increases proportionally with the number of hops in the path. Configuring the DC to be 100% is equivalent to having the radio always on, so the values of *p* are irrelevant in this case. A negative value of *p* implies a decrease of the check interval and an increase of DC.

We used the Avrora simulator [37] to test the applications. Avrora provides a complete simulation framework for evaluating applications written in TinyOS 1.x/2.x over AVR platforms (which is the name of a known hardware platform family) such as Mica2 and MicaZ, and it allows the configuration of two radio models: (1) indoors, which is based on the free space path loss formula; and (2) outdoors, where the user fixes the maximum radio range. We considered the outdoor scenario for our experiments. Table 2 shows the simulation parameters used in our experiments.

**Figure 6.**
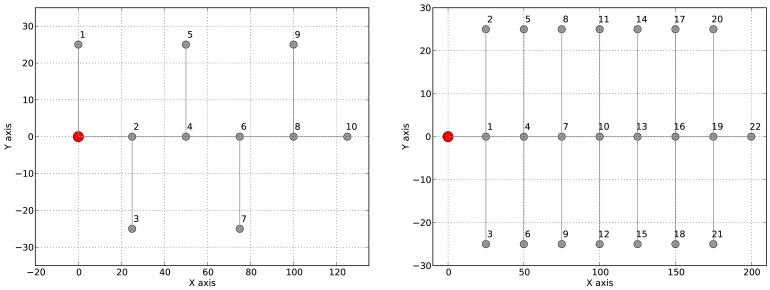
Deployments of 10- and 22-nodes tree topology.

Each application was tested on two different network topologies: (1) two linear topologies of 10 and 20 nodes; and (2) two tree-based topologies as shown in Figure 6, a binary tree of 10 nodes and a tree with 22 nodes where each node has up to three children (see Figure 6 for details). We repeated each experiment 5 times and each experiment took 600 virtual seconds. The 96% confidence interval was at most 0.01 for all experiments. For each experiment we determine the network lifetime, the end-to-end delay and the packet loss ratio. We compare the results with those obtained using CTP/BoX-MAC when no communication delays are accounted for.

### 5.1. Connectivity

In CTP each node in the network sends beacon messages to its immediate neighbors. CTP uses these beacons to construct and maintain routing trees such that a node is not able to communicate if it does not have a parent in the routing tree. Therefore, each node must find a parent with ETX less than ∞. The root is the only node with ETX initialized to 0; network connectivity extends from the root to the leaves. Given this fact, we must ensure that the check intervals do not lead to isolated nodes which yield unconnected networks. This problem can arise for some extremely low values of DC and it becomes worse when the check interval is shifted too much, *i.e.*, when the value of *p* is extremely large. If the value of DC is not appropriate, the activity period in a node may be insufficient to allow CTP to establish a valid route in an instant of time between pairs of nodes if the beacon messages are not received by the neighbors. Note that beacon messages are continuously sent by the nodes during their lifetime, at increasing sending intervals. Thus, although some beacon messages are lost, it does not mean that a node cannot find a route in the future.

**Figure 7.**
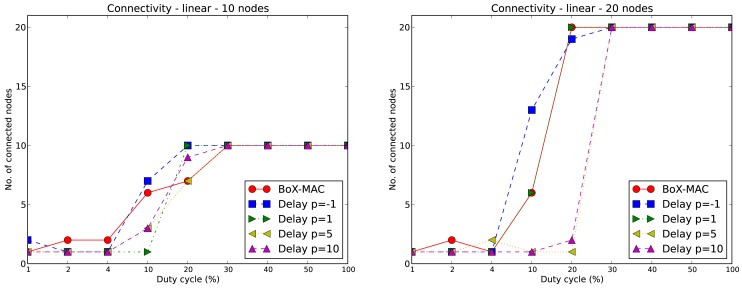
Connectivity of BoX-MAC and CLAC in a 10-node (left) and in a 20-node (right) linear topologies.

We illustrate this issue in Figure 7 by showing the connectivity of CLAC against the connectivity of BoX-MAC for each of the experiments. We measure connectivity by counting the number of nodes successfully linked to a parent. On the left hand side we show the results for a 10-node linear topology using different values of DC and *p*. We observe that for low values of DC (between 1% and 10%) the network is not totally connected for either protocols. For values of DC equal to 20% the network is connected only for the two smallest values of *p* (−1 and 1), while for the two largest values of *p* none of the networks is connected. For values of DC greater than 20% all of the nodes in the network successfully find a valid route to the sink regardless of the strategy. We observe that connectivity is directly proportional to the duty cycle: the larger is the DC, the higher is the connectivity. For negative values of *p* we effectively increase the DC since we reduce the check interval and, as a result, we improve the connectivity with respect to BoX-MAC. On the contrary, for large values of *p* and small DC, the connectivity is low. This is because increasing DC increases the probability of receiving beacon messages, since the beacon messages are sent at arbitrary times (even at times different from the scheduled time for sending the data); when DC is low the beacons may not be received and, consequently, routes may not be determined. The right hand side of Figure 7 shows the connectivity for BoX-MAC and CLAC for a 20-nodes linear topology. The graphic shows a similar trend as the 10-node case. We can observe that the increase in network size results in a lower connectivity in CLAC (for DC ≤ 20%). It is also interesting to note that the increase of DC, when *p* = −1, does not guarantee total network connectivity.

**Figure 8.**
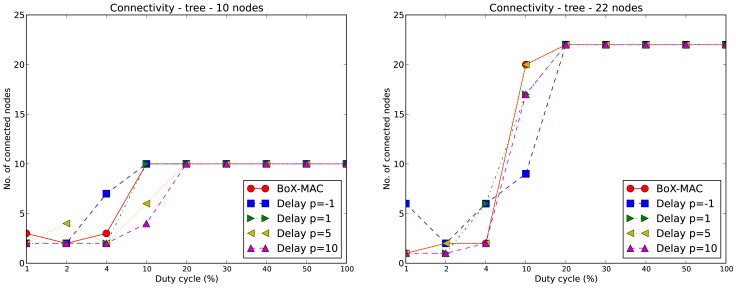
Connectivity of BoX-MAC and CLAC in 10-node (left) and 22-node (right) tree topologies.

Figure 8 shows the connectivity achieved for tree topologies. CLAC improves the connectivity when compared to linear topologies, as observed for DC=20%, for which all networks are completely connected. In the case of the 10-node tree (left hand side) for values of *p* = −1 and *p* = 1 and DC equal to 10%, the network is also connected (as in BoX-MAC), which is an improvement over the linear topology. When we increase the size of the tree (right hand side), the network connectivity decreases slightly for DC = 10%. The reason is that in tree topologies a node is affected by the load of its children; in particular, a heavy load at a node generated by its children may impair its communication with some of them. We can conclude that the neighboring size is an important factor for achieving connectivity, and that the connectivity loss in CLAC as compared to BoX-MAC is mainly due to the loss of beacon messages, whose arrival times cannot be known a priori. Once the topology is established, CLAC may then adapt the delays due to data packets as proposed in this work.

### 5.2. Network Lifetime

**Figure 9.**
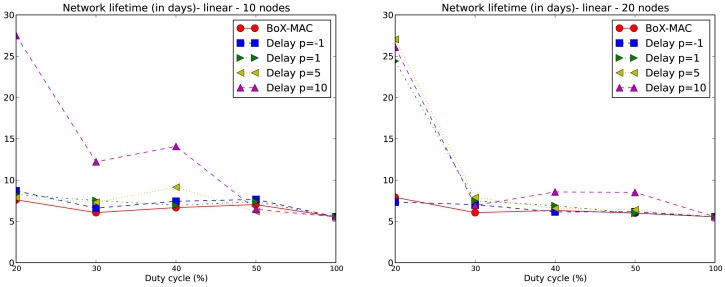
Network lifetime (in days) for 10-node (left) and 20-node (right) linear topologies.

**Figure 10.**
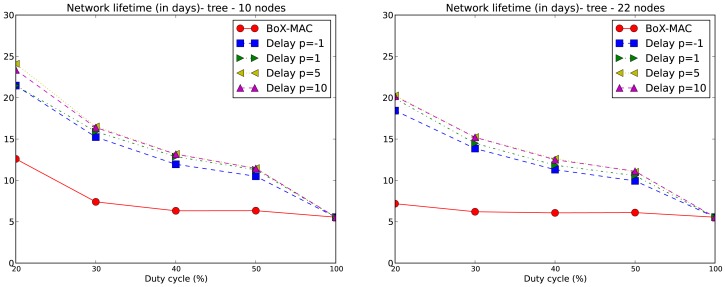
Network lifetime (in days) for 10-node (left) and 22-node (right) tree topologies.

The lifetime of the network is computed for each experiment according to Equation (2). We only consider values of DC above 10% which, based on our experiments, guarantee network connectivity. Note that for DC equal to 20%, network connectivity is guaranteed for tree topologies but not for linear topologies with *p* = 5 and *p* = 10. We compare the network lifetime using CLAC against the lifetime using CTP/BoX-MAC. We express the results of the network lifetime in days. Figure 9 illustrates the results for the 10- and 20-node linear topologies. We observe that the network lifetime decreases when DC increases, which matches the fact that a larger DC implies a bigger energy consumption. With a DC = 100% the lifetime converges to 5.5 days for all experiments. As the figure shows, a larger *p* implies a longer network lifetime. For positive values of *p*, which implies a proportional increase of the delay, we obtain a lower DC and, subsequently, longer network lifetimes. This fact can be observed in Figure 9 where the longest network lifetimes are achieved for *p* = 10. From this figure we observe that CLAC improves the network lifetime when compared to BoX-MAC for all values of DC and *p*. On the right hand side of Figure 9, we see network lifetimes more similar to CTP/BoX-MAC (except for DC = 20%). This behavior is due to the increase in the network size, which causes a bigger load for the forwarding nodes (remember that the network lifetime is the lifetime of the node that first dies). Figure 10 shows the network lifetime for 10- and 22-node tree-based topologies, both of which have a longer lifetime compared to linear topologies. In a linear topology, the node which is the nearest to the sink must receive N−1 packets and transmit N packets in each round, being N the number of nodes in the linear topology. It also accumulates the delays produced by each hop, which leads to high idle listening and consequently a lot of retransmissions. It follows that node 1 in a linear topology is very overloaded trying to receive and retransmit many packets, while, in the case of the tree, the overall traffic is split among the children of the root. Therefore, node 1 dies in a linear topology much earlier than in a tree topology. With a low DC the situation gets worse since the contention increases. For tree-based topologies the lifetime is increased, and CLAC doubles the lifetime obtained by CTP/BoX-MAC for some values of DC and *p*.

### 5.3. Packet Loss

**Figure 11.**
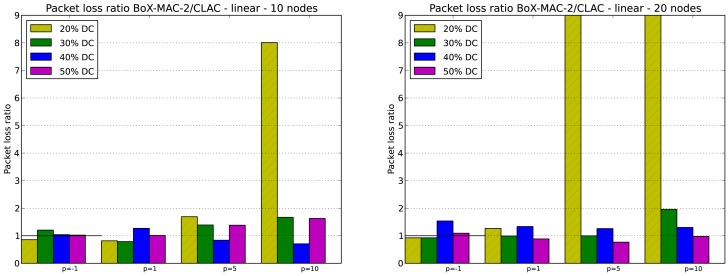
Packet loss ratio for 10- and 20-node linear topologies.

**Figure 12.**
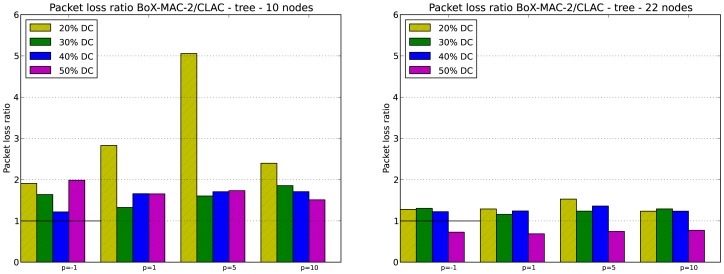
Packet loss ratio for 10- and 22-node tree topologies.

We evaluate the packet loss in CLAC for DC greater or equal to 20% which, based on our experiments, guarantee network connectivity. As in the case of network lifetime, keep in mind that for DC equal to 20% network connectivity is guaranteed for tree topologies but not for linear topologies with *p* = 5 and *p* = 10. Specifically, we evaluate the packet loss ratio, which is defined as the ratio between the number of packets lost in CTP/BoX-MAC over the number of packets lost in CLAC. Figures 11 and 12 illustrate the packet loss ratio for different values of *p*; when this ratio is greater than 1.0 it indicates that there was more packet loss in CTP/BoX-MAC than in CLAC.

Figure 11 shows the packet loss ratio for the 10- and 20-node linear topologies. The packet loss ratio indicates that CLAC reduces the number of lost packets for most DC. Note that for *p* = 5 or *p* = 10, and DC = 20% the packet loss ratio is very high. This behavior coincides with the cases when the experiments do not yield a completely connected network. The lack of connectivity causes a high packet loss. According to the results, we can conclude that for DC greater or equal to 20%, which maintain the network connectivity and provide energy savings, CLAC may reduce packet loss. However, the optimal value of *p* that provides the minimum packet loss is highly dependent on DC, the topology, and the network size.

Figure 12 shows the packet loss ratio for 10- and 22-node tree topologies. CLAC significantly reduces the number of packets that are lost as compared to CTP/BoX-MAC, for all values of DC and *p*, with the exception of DC = 50% in the 22-node tree topology. If we increase both the size of the network and the DC, the load of the network grows and the packet loss increases. We conclude that CLAC performs better in scenarios with DC less than 50%.

### 5.4. End-to-end Delay

In order to show that CLAC does not increase the end-to-end delay, we measure the end-to-end delay for CLAC and BoX-MAC. First, we consider the 10-node linear topology where node 10 is the only data source and, second, we consider the 10-node tree topology with multiple sources: node 5, 7 and 9.

**Figure 13.**
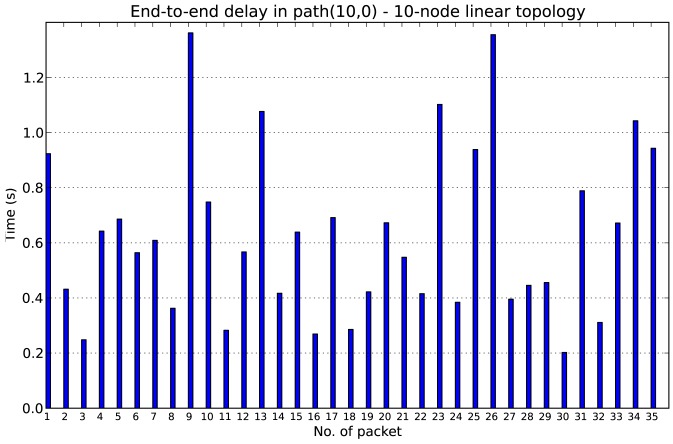
End-to-end delay between node 10 and 0 in a 10-node linear topology with DC = 30%.

In the 10-node linear topology, node 10 sends a data packet to node 0 (the sink) every 10 seconds. Within *path*(10, 0) node 10 is 10 hops away from node 0. All the packets travel along the same path to achieve the destination, *i.e.*, there is no changes in the topology. Figure 13 shows the end-to-end delay between node 10 and node 0 obtained for the linear topology with DC = 30%. The time that a packet takes from the source to the destination is the end-to-end delay, which we compute as the difference of the time of transmission and the reception. Each packet takes a variable amount of time to reach node 0, due to the effect of the retransmissions and the accumulated latencies.

**Figure 14.**
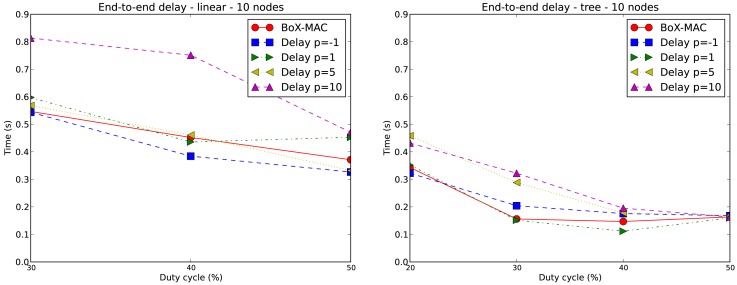
End-to-end delay for 10-node linear and 10-node tree topologies.

Figure 14 on the left presents the average end-to-end delay for a 10-node linear topology with only a source (node 10) for DC = 30%, 40% and 50%. We exclude the case of DC equal to 20% since it does not guarantee complete connectivity in the network. On the right hand side, we show the end-to-end delay for a 10-node tree topology for DC greater or equal to 20%, where nodes 5, 7 and 9 are data sources. Thus, we have: *path*(5, 0) = {5, 4, 2, 0}, *path*(7, 0) = {7, 6, 4, 2, 0} and *path*(9, 0) = {9, 8, 6, 4, 2, 0}. We show the average end-to-end delay within *path*(9, 0), which is the worst case.

In general when DC increases, the end-to-end delay is reduced, which matches the fact that a larger DC implies a lower contention. Even in the case where there is no contention due to the fact of having one single source (as represented by Figure 14 on the left), the end-to-end delay grows as a result of having strict duty cycles, *i.e.*, we reduce the opportunity for fast forwarding and hence the penalty on the end-to-end delay. Note that when *p* increases the end-to-end delay also increases, since it is equivalent to reducing the DC. The end-to-end delay in the linear topology is larger than in the tree topology because of the difference in the path lengths, which is 10 hops for the linear topology and 5 hops in the tree topology. The average end-to-end delay of BoX-MAC is for most of the times between the end-to-end delay of CLAC with *p* = − 1 and *p* = 1. This result is within expectation since the DC used in BoX-MAC is above the DC used in CLAC with *p* = −1 and below the DC used in CLAC with *p* = 1. It follows that the end-to-end delays depend on the duty cycles used and, correspondingly, present the same trend.

## 6. Conclusions

The use of duty cycles to govern the radio activity of the nodes is extremely useful to limit their energy consumption. Preamble sampling-based protocols efficiently address this task by providing schemes to schedule the time at which the communicating nodes must turn their radio transceivers on and off. However, due to channel access contention and the internal processing (among other reasons), the times of radio activation in the sender and receiver may not match. A good synchronization strategy is essential to saving the node resources.

In this paper we propose an approach to deal with communication delays which occur in preamble sampling-based protocols in order to improve the synchronization of the nodes. Our approach, called Cross Layer Adaptation of Check intervals (CLAC), allows to precisely update the check interval at each hop based on estimating the delay that a packet traveling along a multi-hop path introduces at each hop within the path. CLAC successfully synchronizes the sender and the receiver by delaying the check interval to match the expected packet arrival time. To estimate the check interval, CLAC uses information provided by the routing and MAC layers. We have shown by simulation that for the linear and tree topologies, CLAC improves the synchronization between the nodes and contributes to finding a good balance between connectivity and energy saving at no additional packet loss. CLAC increases the network lifetime, maintains the connectivity and reduces the packet loss as compared to CTP/BoX-MAC. Thus, we demonstrate that for dealing with network latencies, it is not necessary to increase the duty cycle and the radio activation in the nodes can be adapted to suit this need.

Ongoing work includes the extension of CLAC to other LPL protocols beyond BoX-MAC. We also plan to analyze the behavior of CLAC on different network topologies (for example grids) in order to study the delays that the packets traveling from the sender to the sink introduce and how they are propagated. Finally, we also plan to compare CLAC with other approaches that adjust the duty cycle in order to find a good balance between high packet delivery and energy savings.

## Figures and Tables

**Table 1.** Configuration of delays for different values of DC and *p*. The values of delays and *τ_new_check_* are all expressed in milliseconds.

**DC** **(%)**	***τ_check_*** **(ms)**	***p* = −1**		***p* = *1***		***p* = *5***		***p* = 10**	

		**Delay**	*τ_new-check_*	**Delay**	*τ_new-check_*	**Delay**	*τ_new-check_*	**Delay**	*τ_new-check_*
1	495	−4.95	490.05	4.95	499.95	24.75	519.75	49.5	544.5
2	245	−2.45	242.55	2.45	247.45	12.25	257.25	24.5	269.5
4	120	−1.2	118.8	1.2	121.2	6	126	12	132
10	45	−0.45	44.55	0.45	45.45	2.25	47.25	4.5	49.5
20	20	−0.2	19.8	0.2	20.2	1	21	2	22
30	11.6	−0.11	11.49	0.11	11.71	0.58	12.18	1.16	12.76
40	7.5	−0.075	7.425	0.075	7.575	0.375	7.875	0.75	8.25
50	5	−0.05	4.95	0.05	5.05	0.25	5.25	0.5	5.5
100	0	–	0	–	0	–	0	–	0

**Table 2.** Parameter values used in the simulations.

**Parameter**	**Value**
Bit rate	250 Kbps
Radio range	30 m
Rx current	18.8 mA
Tx current	17.4 mA (at 0 dBm)
Sending interval	10 s
Data length	40 B
Battery capacity	2500 mAh
Voltage	3 v
DUTY_ON_TIME^2^	5 ms

We assume DUTY_ON_TIME equal to 5 ms as defined in BoX-MAC-2.
